# Carrot Intake and Risk of Developing Cancer: A Prospective Cohort Study

**DOI:** 10.3390/nu15030678

**Published:** 2023-01-29

**Authors:** Ulrik Deding, Gunnar Baatrup, Lasse Kaalby, Morten Kobaek-Larsen

**Affiliations:** 1 Department of Clinical Research, University of Southern Denmark, 5000 Odense, Denmark; 2 Department of Surgery, Odense University Hospital, 5000 Odense, Denmark

**Keywords:** falcarinol, falcarindiol, polyacetylenes, carrots, cancer, adenocarcinoma, primary prevention

## Abstract

A prospectively followed Danish cohort of 55,756 citizens with an observation time upwards of 25 years was investigated for association between eating raw carrots on a regular basis and developing various adenocarcinoma-dominant cancers and leukemia. Mean age at inclusion was 56.2 years (SD 4.4 years), and 52% were females. A dose-dependent reduction in incidence was seen for cancer of the lung (HR 0.76, CI95% 0.66; 0.87) and pancreas (HR 0.79, CI95% 0.61; 1.03), as well as leukemia (HR 0.91, CI95% 0.68; 1.21). Only for lung cancer was the association significant. In the case of pancreatic cancer, a possible type 1 error was present due to a low number of cancers. In cases of breast and prostate cancer, no association and no dose response were demonstrated. The association seen for lung and pancreatic cancer parallels that earlier demonstrated for large bowel cancer and indicates a cancer-protective effect from daily intake of raw carrots not limited to gastrointestinal adenocarcinomas. Processed carrots exhibited no effect. The preventive effect could be due to the polyacetylenic compounds falcarinol and falcarindiol in carrots, whereas carotene may not have an effect. The polyacetylenes are inactivated by heating, supporting our findings that only raw carrot intake has an effect. Indirect evidence for the cancer preventive effect of carrots in humans has reached a level where a prospective human trial is now timely.

## 1. Introduction

The association between consistent intake of fruits and vegetables and a reduced risk of developing some types of cancer has been shown in cohort studies for many decades [1,2,3]. The association between a low risk of bowel cancer and high consumption of carrots has attracted special interest both because the association has been shown repeatedly in very large cohorts with extended confounder control [4,5], as well as because the effect is marked with a preventive potential as high as population-based screening programs. Large bowel cancer is the third most common cause of cancer deaths, and the potential effect on population health of increasing the consumption of raw carrots is marked. Further, some studies have shown a negative correlation between carrot intake and development of other cancers dominated by the adenocarcinoma type such as breast, lung, gastric, and prostate cancers [5]. For many years, the health-promoting effect of carrots has been attributed to a high content of beta-carotene in orange carrots because of its potent antioxidant capacity. This has been disproven by randomized trials administering purified beta-carotene to healthy citizens [6,7,8,9,10,11]. Some studies actually demonstrated increased incidence of lung cancer in those taking beta-carotene [12,13]. 

Carrots are the major dietary source of the falcarinols—falcarinol and falcarindiol—and their interaction with animals and human cancer cells and enzyme systems have been systematically investigated [14]. Purified falcarinols have been demonstrated to inhibit the growth of human cancer cell lines [15,16,17,18,19] and prevent neoplastic transformation in the large bowel in rats primed to develop bowel cancer [20,21,22]. The effect seen in rats can be reproduced when feeding the rats with raw carrots with purified falcarinols. This cancer preventive effect can be explained by the anti-inflammatory effect of the falcarinols demonstrated in several different experimental set-ups [21,23,24] and is believed to be, at least in part, mediated by an anti-inflammatory effect very similar to the cancer-preventive effect provided by aspirin and other pharmacological inhibitors of inflammation. It therefore seems obvious to investigate an additional effect in other cancers of the adenocarcinoma type. Different cultivars vary markedly in the polyacetylenic content, and awareness of this and a change in carrot cultivar available to the citizens might have a positive effect.

As an extension of our formerly published article on bowel cancer incidence [4], we aimed to estimate the risk of adenocarcinoma-dominated cancer types (lung, breast, prostate, and pancreas) on the basis of carrot intake, and we included leukemia as a control.

## 2. Materials and Methods

### 2.1. Population

This was a prospective cohort study investigating risk of leukemia, breast cancer, lung cancer, prostate cancer, and pancreatic cancer in individuals originally included in the Diet, Cancer and Health study (DCH). The cohort was linked with National Danish registers in order to identify diagnoses of the diseases of interest in the years to come. 

DCH invited a sample (n = 160,725) of Danish born residents of the Aarhus and Copenhagen areas aged 50 to 64 years of age to participate. Non-responders were reminded after three weeks, and again after six weeks. The inclusion period lasted from 1993 until 1997. All participants (n = 57,053) filled out a validated 192 item food frequency questionnaire, describing their dietary intake during the preceding 12 months [25,26,27]. Additionally, a questionnaire collecting lifestyle data on known cancer risks (smoking, alcohol, physical activity, etc.) was filled in by participants. Individuals underwent an examination in which their height and weight were measured by a lab technician [25]. A group of participants (n = 585) were excluded from the original cohort as they had a prior colorectal cancer diagnosis [4], thereby fulfilling the DCH exclusion criteria. We followed each individual in the national registers from their date of inclusion until death, 31 December 2018, or diagnosis, whichever came first.

### 2.2. Register Data

The DCH cohort was linked with the Danish National Patient Register [28] in order to identify ICD-8 (1985–1993) and ICD-10 (1994–2018) codes (detailed in Table 1) of the outcomes of interest. ICD-8s were included in order to identify diagnoses prior to inclusion, enabling us to exclude those individuals. The Danish Register of Causes of Death [29] was used to identify dates of death.

### 2.3. Exposure

Dietary intake of raw carrot was the main exposure of interest. Additionally, intake of processed carrot was also investigated. Both were estimated from the self-reported data in the food frequency questionnaires. Processed carrots are of less interest as the levels of FaOH and FaDOH in carrots decrease when they are thermally processed. In this cohort, we differentiated between consumption of raw and processed carrots. Falcarinols are sensible to heat treatment, and the activity has been shown to decrease after heating [30]. Thermal processing of carrots at 90 °C for 2 min will reduce biological activity by 25–50% [31], but the minimal active dose necessary to conduct biological effect is largely unknown. Participants would report their frequency of raw and processed carrot intake ranging from never to eight or more times per day. This was translated to gram per day (g/day) using standard portion sizes and categorized into no intake, less than 32 g/day, and more than 32 g/day of raw and processed carrot. This division was chosen on the basis of the evidence available from rodent studies, and it was this categorization used to identify significant differences in risk of colorectal cancer in the cohort as described previously [4]. 

### 2.4. Outcomes

Outcomes were defined as any diagnose of leukemia, breast cancer, prostate cancer, pancreatic cancer, or lung cancer registered in the Danish National Patient Register after the individual date of inclusion. Specific ICD-8 and ICD-10 codes are provided in Table 1.

### 2.5. Statistical Analysis

Baseline characteristics were compared using chi-squared tests. The relative risk of the respective diseases was estimated between groups defined by carrot intake (raw and processed) using Cox proportional hazard regression models adjusting for a number of relevant covariates (detailed in Section 2.6). The level of significance was set at 5%, and 95% confidence intervals (CI95%) were provided. Interactions between raw carrot intake and covariates were tested in all models but were only significant for age group in prostate cancer analyses. Therefore, the regression model for prostate cancer was conducted using age as time, comparing individuals of the same age instead of adjusting for age group at entry as a covariate in the regression model. This method was also performed for the other outcomes as a sensitivity analysis to ensure the conclusions would not change. Cumulative incidence proportion curves stratified by raw carrot intake were created for the outcome of lung cancer. Data management was performed using SAS software version 9.4 (SAS Institute Inc. SAS 9.4. Cary, NC, USA), and statistical analyses were conducted using R statistical software package version 3.6.1 (R Core Team, Vienna, Austria) [32,33,34].

### 2.6. Covariates

The Cox proportional hazards regression models were adjusted for sex, age group, metabolic equivalents (MET) score, other vegetable intake, other root vegetable intake, intake of non-steroidal anti-inflammatory drugs (NSAID), smoking status, educational level, alcohol consumption, body mass index (BMI), former cerebral or coronary artery thrombosis, and hormone replacement therapy. Except for BMI, all covariates relied on self-reported data.

All covariates were included as categorical variables. Sex was defined as male or female. Age groups were divided as 50–54, 55–59, and 60–65 years of age (at baseline). MET score, other vegetable consumption (spinach, salad, cucumber, bell pepper, eggplant, tomato, squash, avocado, beans, and peas), and other root vegetable consumption (celery, ginger root, and partly frozen carrot from vegetable mix) [4] was divided into quartiles of quantity within the sample. NSAID consumption was divided into consumer or non-consumer. Smoking status was grouped into non-smoker, former smoker, and current smoker. Educational level was grouped as low, medium, and high level of education. Alcohol consumption was determined on the basis of the number of units consumed per week and grouped according to the Danish guidelines on maximum consumption of ten units per week, i.e., none, 1–10 units per week, and more than 10 units per week. BMI was grouped as below 18.5, 18.5–25, and above 25. Former cerebral or coronary artery thrombosis were categorized as yes or no.

## 3. Results

**Figure 1:**
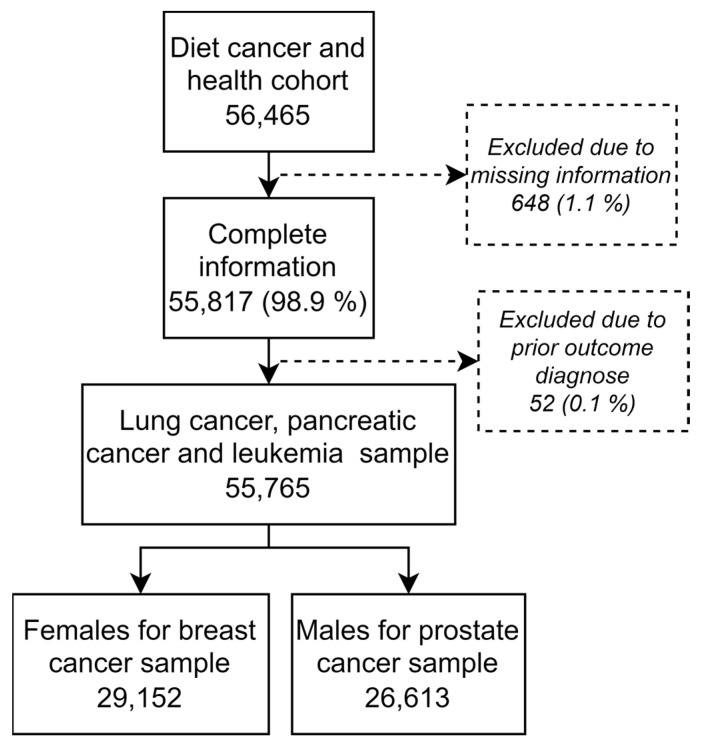
Flow of participants including sample size for each outcome measure.

The DCH cohort (n = 56,465) was linked to national registers, and individuals with missing information (n = 648) and individuals with prior outcome diagnosis (n = 52) were excluded. This left 55,765 individuals for analysis of lung cancer risk, pancreatic cancer risk, and leukemia risk, and 29,152 (52%) females for analysis of breast cancer risk and 26,613 (48%) males for prostate cancer risk analysis (Figure 1). Mean age at inclusion was 56.2 years (standard deviation (SD) 4.4 years). The mean follow-up time varied from 20.0 years (SD 4.9) in prostate cancer analyses to 21.1 years (SD 3.9) in lung and pancreatic cancer analyses. Number of cases registered were 542 (1.0%) for leukemia, 2451 (4.4%) for lung cancer, 655 (1.2%) for pancreatic cancer, 2719 (9.3%) for breast cancer, and 2821 (10.6%) for prostate cancer (Table 2). Individuals who died prior to each specific diagnosis and thereby lost to follow-up were 16,625 (29.8%) for leukemia, 15,065 (27.0%) for lung cancer, 16,423 (29.5%) for pancreatic cancer, 6638 (22.8%) for breast cancer, and 8166 (30.7%) for prostate cancer.

The disease incidence proportion was significantly higher in those who did not eat any raw carrots for the outcomes of leukemia, lung cancer, pancreatic cancer, breast cancer, and prostate cancer compared to carrot eaters. Regarding processed carrot intake, this was only the case for lung cancer incidence (Table 2). Baseline covariate distributions in the sample has been provided in Supplementary Table S1.

**Figure 2:**
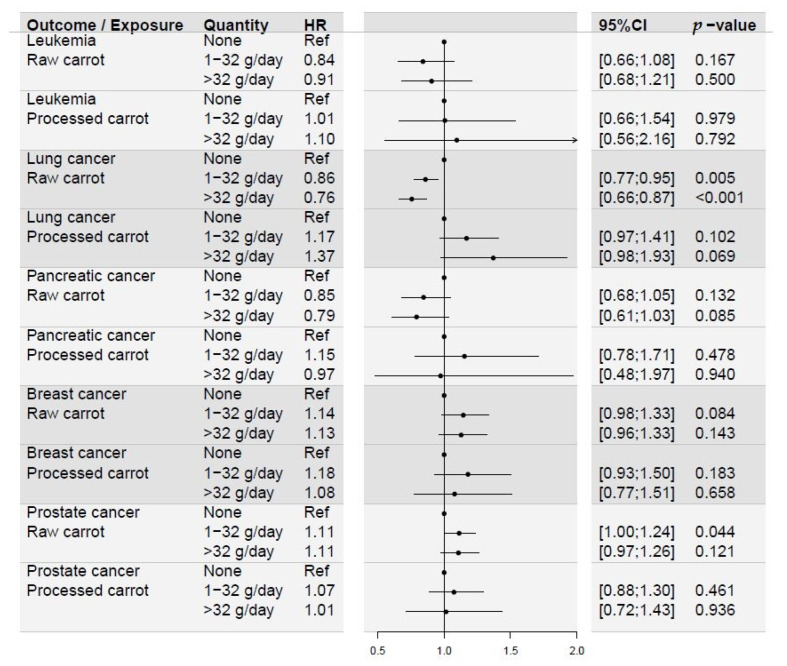
Forest plot illustrating the results from multivariate Cox proportional hazards regression models estimating the risk of the outcomes listed on the basis of carrot intake. All models were adjusted for sex, age group, MET score, other vegetable consumption, other root vegetable consumption, NSAID intake, smoking status, educational level, alcohol consumption, BMI, and former cerebral or coronary artery thrombosis. Breast cancer analysis was additionally adjusted for hormone replacement therapy. Leukemia, lung cancer, and pancreatic cancer, n = 55,765. Breast cancer, n = 29,152. Prostate cancer, n = 26,613.

**Figure 3:**
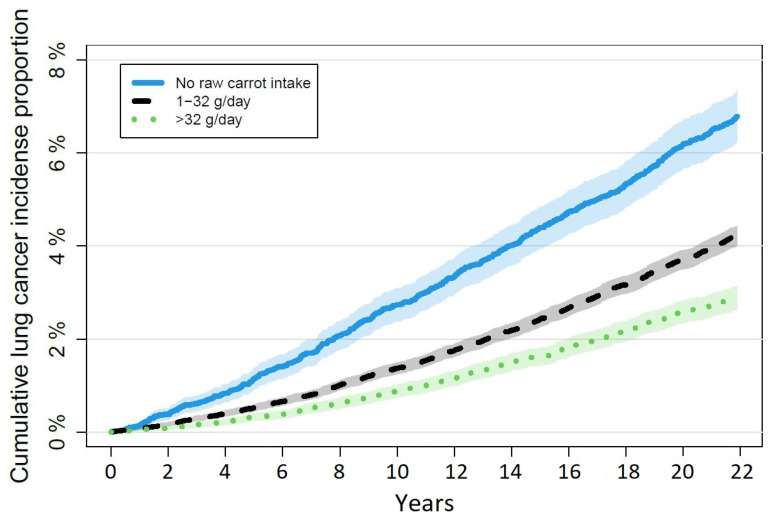
Cumulative incidence proportions of lung cancer during follow-up stratified by raw carrot intake. n = 55,765.

The adjusted hazard ratios (HR) from the Cox proportional hazards regression models revealed significant differences in risk of lung cancer and prostate cancer on the basis of raw carrot intake. The HR for lung cancer were 0.86 (CI95% 0.77; 0.95) in the group eating 1–32 g/day and 0.76 (CI95% 0.66; 0.87) in the group eating more than 32 g/day, compared to those eating no raw carrots. The HR for prostate cancer was significantly increased at 1.11 (CI95% 1.00; 1.24) in the group eating 1–32 g/day, and increased, yet not statistically significantly, at 1.11 (CI95% 0.97; 1.26) in the group eating more than 32 g/day, compared to those eating no raw carrots. Processed carrot intake did not show the same effect (Figure 2). The cumulative incidense proportion curves for lung cancer stratified by raw carrot intake is illustrated in Figure 3. For leukemia, breast cancer, and pancreatic cancer, there were no significant differences in risk of disease during follow-up on the basis of neither raw nor processed carrot intake. Processed carrot intake did not affect prostate cancer risk either. Although the trends showed HR estimates above one for prostate and breast cancer risk according to increasing carrot intake, the pattern in pancreatic cancer and leukemia were similar to that of lung cancer (Figure 2).

The sensitivity analyses conducting the regression models using age as time for outcomes of leukemia, breast cancer, lung cancer, and pancreatic cancer did not alter the conclusion of the main analyses.

## 4. Discussion

This cohort of 57,000 Danish citizens was followed for more than 20 years through extended questionnaires and clinical controls [25]. We have shown earlier that citizens eating raw carrots have reduced risk of developing colorectal cancer [4]. This was true also after confounder corrections in multivariate test; it was dose dependent and amounted to a 17% risk reduction in the high exposure group. Two hypotheses arose from this observation: (1) Can this preventive effect be reproduced in other types of common human cancer diseases, especially adenocarcinomas as indicated in the literature. (2) Is this effect counterbalanced by a possible carcinogenic effect of beta-carotene in the case of lung cancer. Leukemia was included in the study as a non-carcinomatous cancer control [35]. The design of the study including confounder control is parallel to our earlier published article on colorectal cancer in this population to allow for comparison.

Even though the population is large and the observational period is long, sufficiently high prevalence of cancer diseases allow only for analysis of the more common types. Even in the cases of leukemia and pancreatic cancer, the numbers allow for only limited subgroup analysis. 

We find no general pattern of the effect of raw and/or processed carrot intake between the different types of cancer. There is a reduced prevalence in the cancers of the lung and pancreas, as well as leukemia. Although the HR is comparable in these three types, the difference is only significant in the case of lung cancer, which also expresses a convincing dose response, as indicated in Figure 3. The HR of the group of lung cancer patients with high exposure to raw carrots show an even higher preventive effect on cancer prevention than what was seen in colorectal cancer. The dose dependency in both leukemia and pancreatic cancer might indicate a true effect of raw carrots in these cancer types as well, even though the differences are not significant. The total prevalence is much lower in leukemia and pancreatic cancer as compared to lung cancer. This might explain the non-significance in the former two.

The correlation between eating carrots and the incidence of the hormone-influenced cancers of the breast and prostate is different. Overall, there is no significant correlation between eating raw or processed carrots and the incidence. The only significant difference is for the low dose of raw carrots in case of prostate cancer. Indeed, in all groups of raw and processed carrot intake for both prostate and breast cancer, it seems as if carrots increase the risk of cancer. The absence of a positive effect in breast cancer is surprising and in contrast to the general findings in earlier cohort studies [36]. It is not easily explainable, even though we assume that the net effect is a balance between the falcarinols and that the concentration of these substances vary widely between different cultivars. The prevalence of breast cancer is high, and a simple type 2 error seems unlikely.

As discussed previously [4], we estimated the cancer risks on the basis of self-reported recall data with an inherent risk of recall bias and healthy food consumption overestimation. If overestimation of carrot intake systematically (or even if random over- and under-estimation) has occurred, the effects seen in our study are probably underestimated. Further, the type of carrot and specific handling were not registered, and it is possible that even greater effects could be achieved by excluding intake of carrots low in falcarinols, or if we had been able to register the intake throughout follow-up instead of limited to the year prior to inclusion. Individuals eating carrots may also make other healthy lifestyle choices more often than their peers, introducing risk of confounding factors, although the extensive adjustments for health-related covariates should limit this risk. Whether the effects seen in this cohort can be transferred to high-risk individuals, such as those with gene mutations or family history of adenocarcinoma, is unknown. The impact of our findings would increase if such populations benefit in the same way or to an even higher degree. Although cohort studies in general have an inherited risk of confounder-driven misinterpretation, the data confirm other studies from different cohorts. These findings should be confirmed in a prospective randomized trial, but this will be very expensive and time consuming because the number needed to include will be in the thousands and observation period has to be for decades.

## 5. Conclusions

Our study confirms earlier studies showing that consistent intake of raw carrots protects against cancers of the lung, as it does in the large bowel. We interpret the results as indicative of a similar effect in pancreatic cancer and leukemia.

## Figures and Tables

**Table 1:** ICD-8 and ICD-10 codes utilized to identify diagnoses of outcomes.

Disease	ICD-8	ICD-10
Breast cancer	174.00; 174.01; 174.02; 174.08; 174.09	C50
Lung cancer	162.09-19	C33; C34; C45
Prostate cancer	185.99	C61
Pancreatic cancer	157.09; 157.80; 157.81; 157.89; 157.99	C25
Leukemia	204.09–207.99	C91–C95

**Table 2:** Baseline carrot intake by outcomes during follow-up.

		**No Leukemia, n = 55,223 (%)**	**Leukemia During Follow-Up, n = 542 (%)**	**Total, n = 55,765**	***p*-Value**
Raw carrot intake	None	7800 (98.8)	93 (1.2)	7893	
	1–32 g/day	31,199 (99.1)	294 (0.9)	31,493	
	>32 g/day	16,224 (99.1)	155 (0.9)	16,379	0.130
Processed carrot intake	None	2240 (98.9)	24 (1.1)	2264	
	1–32 g/day	51,699 (99.0)	503 (1.0)	52,202	
	>32 g/day	1284 (98.8)	15 (1.2)	1299	0.715
		**No Lung Cancer, n = 53,314 (%)**	**Lung Cancer During Follow-Up, n = 2451 (%)**	**Total, n = 55,765**	***p*-Value**
Raw carrot intake	None	7336 (92.9)	557 (7.1)	7893	
	1–32 g/day	30,097 (95.6)	1396 (4.4)	31,493	
	>32 g/day	15,881 (97.0)	498 (3.0)	16,379	<0.001
Processed carrot intake	None	2138 (94.4)	126 (5.6)	2264	
	1–32 g/day	49,930 (95.6)	2272 (4.4)	52,202	
	>32 g/day	1246 (95.9)	53 (4.1)	1299	0.019
		**No Pancreatic Cancer, n = 55,110 (%)**	**Pancreatic Cancer During Follow-Up, n = 655 (%)**	**Total, n = 55,765**	***p*-Value**
Raw carrot intake	None	7773 (98.5)	120 (1.5)	7893	
	1–32 g/day	31,123 (98.8)	370 (1.2)	31,493	
	>32 g/day	16,214 (99.0)	165 (1.0)	16,379	0.002
Processed carrot intake	None	2236 (98.8)	28 (1.2)	2264	
	1–32 g/day	51,587 (98.8)	615 (1.2)	52,202	
	>32 g/day	1287 (99.1)	12 (0.9)	1299	0.675
		**No Breast Cancer, n = 26,433 (%)**	**Breast Cancer During Follow-Up, n = 2719 (%)**	**Total, n = 29,152**	***p*-Value**
Raw carrot intake	None	2324 (92.1)	198 (7.9)	2522	
	1–32 g/day	13,825 (90.4)	1472 (9.6)	15,297	
	>32 g/day	10,284 (90.7)	1049 (9.3)	11,333	0.017
Processed carrot intake	None	846 (92.4)	70 (7.6)	916	
	1–32 g/day	24,826 (90.6)	2572 (9.4)	27,398	
	>32 g/day	761 (90.8)	77 (9.2)	838	0.201
		**No Prostate Cancer, n = 23,792 (%)**	**Prostate Cancer During Follow-Up, 2821 (%)**	**Total, n = 26,613**	***p*-Value**
Raw carrot intake	None	4869 (90.7)	502 (9.3)	5371	
	1–32 g/day	14,433 (89.1)	1763 (10.9)	16,196	
	>32 g/day	4490 (89.0)	556 (11.0)	5046	0.004
Processed carrot intake	None	1228 (91.1)	120 (8.9)	1348	
	1–32 g/day	22,152 (89.3)	2652 (10.7)	24,804	
	>32 g/day	412 (89.4)	49 (10.6)	461	0.115

## Data Availability

Not applicable.

## Supplementary Materials

The following supporting information can be downloaded at: https://www.mdpi.com/article/10.3390/nu15030678/s1, Supplementary Table S1. Baseline covariate distributions in the sample.
