# Supplementary material for: Carrot Intake and Risk of Developing Cancer: A Prospective Cohort Study
Source: Nutrients. 2023 Jan 29;15(3):678. doi: 10.3390/nu15030678 (PMC9919376; doi:10.3390/nu15030678)
Supplement: Supplementary file 1 [file nutrients-15-00678-s001.zip › nutrients-2139106-supplementary.pdf]

Supplementary Table S1. Baseline covariate distributions in the sample, n=55,765

Variable	Subgroup	No diagnose during follow-up, n=46,878 (%)	Diagnosed during follow-up, n=8,887 (%)	Total, n=55,765	p-value
Sex	Female	24,896 (85.4)	4,256 (14.6)	29,152	
	Male	21,982 (82.6)	4,631 (17.4)	26,613	<0.001
Age group	50 - 54	20,366 (86.4)	3,216 (13.6)	23,582	
	55 - 59	14,382 (83.3)	2,880 (16.7)	17,262	
	60 - 64	12,130 (81.3)	2,791 (18.7)	14,921	<0.001
MET score	1st quartile	11,883 (83.8)	2,298 (16.2)	14,181	
	2nd quartile	12,001 (84.4)	2,224 (15.6)	14,225	
	3rd quartile	11,377 (84.1)	2,144 (15.9)	13,521	
	4th quartile	11,617 (83.9)	2,221 (16.1)	13,838	0.588
Other vegetable consumption	1st quartile	11,490 (82.7)	2,399 (17.3)	13,889	
	2nd quartile	11,689 (83.8)	2,267 (16.2)	13,956	
	3rd quartile	11,823 (84.9)	2,108 (15.1)	13,931	
	4th quartile	11,876 (84.9)	2,113 (15.1)	13,989	<0.001
Other root vegetable consumption	1st quartile	11,489 (82.6)	2,412 (17.4)	13,901	
	2nd quartile	11,761 (84.4)	2,176 (15.6)	13,937	
	3rd quartile	11,733 (84.0)	2,229 (16.0)	13,962	
	4th quartile	11,895 (85.2)	2,070 (14.8)	13,965	<0.001
NSAID	Non-consumer	31,637 (84.2)	5,945 (15.8)	37,582	
	Consumer	15,241 (83.8)	2,942 (16.2)	18,183	0.280
Smoking status	Non-smoker	16,999 (87.0)	2,545 (13.0)	19,544	
	Former smoker	13,716 (85.2)	2,376 (14.8)	16,092	
	Current smoker	16,163 (80.3)	3,966 (19.7)	20,129	<0.001
Educational level	Low	15,208 (82.9)	3,129 (17.1)	18,337	
	Medium	21,760 (84.6)	3,957 (15.4)	25,717	
	High	9,910 (84.6)	1,801 (15.4)	11,711	<0.001
Alcohol consumption	None	1,086 (84.3)	203 (15.7)	1,289	
	1-10 units per week	27,629 (84.8)	4,939 (15.2)	32,568	
	>10 units per week	18,163 (82.9)	3,745 (17.1)	21,908	<0.001
Body mass index	<18.5	395 (83.5)	78 (16.5)	473	
	18.5 - 25	20,158 (83.8)	3,889 (16.2)	24,047	
	>25	26,325 (84.3)	4,920 (15.7)	31,245	0.377
Former cerebral or coronary artery thrombosis	No	45,399 (84.1)	8,604 (15.9)	54,003	
	Yes	1,479 (83.9)	283 (16.1)	1,762	0.911
